# Contributions of coxsackievirus adenovirus receptor to tumorigenesis

**DOI:** 10.1042/BST20221203

**Published:** 2023-06-12

**Authors:** Claudia Owczarek, Yassmin Elmasry, Maddy Parsons

**Affiliations:** Randall Centre for Cell and Molecular Biophysics, King's College London, Guys Campus, London SE1 1UL, U.K.

**Keywords:** cancer, cell adhesion, signalling

## Abstract

Coxsackievirus and adenovirus receptor (CAR) is a transmembrane cell–cell adhesion receptor that forms homodimers across junctions and plays a key role in mediating epithelial barrier integrity. CAR can also heterodimerise with receptors on the surface of leukocytes and thus plays an additional role in mediating immune cell transmigration across epithelial tissues. Given the importance of both biological processes in cancer, CAR is emerging as a potential mediator of tumorigenesis as well as a target on cancer cells for viral therapy delivery. However, the emerging, often conflicting, evidence suggests that CAR function is tightly regulated and that contributions to disease progression are likely to be context specific. Here, we summarise reported roles for CAR in the context of cancer and draw on observations in other disease settings to offer a perspective on the potential relevance of this receptor as a therapeutic target for solid tumours.

## Introduction

The coxsackie and adenovirus receptor (CAR) was first identified and isolated as a viral receptor for the coxsackie B virus (CVB) and shortly after for adenovirus (Ad) serotypes 2 and 5 [1,2]. CAR is a 46 kDa transmembrane glycoprotein belonging to the junction adhesion molecule (JAM) family within the immunoglobulin superfamily (IgSF) [3]. Other key IgSF members showing high homology to CAR include endothelial cell-selective adhesion molecule (ESAM; [4]), BT-IgSF [5] and CAR-Like Membrane Protein (CLMP; [6]), where all these proteins share a common feature in their localisation to cell–cell adhesions. Subsets of JAM proteins are also expressed on the surface of leukocytes, platelets, and erythrocytes, and epithelial CAR can form heterodimers with a several of these receptors to mediate epithelial–immune cell interactions [7]. Thus, CAR plays an important role in epithelial tissue homeostasis and tissue inflammation and emerging evidence supports a role for CAR in regulating initiation and progression of a range of different pathologies, including cancer.

## CAR structure, localisation and binding partners

**Figure 1.**
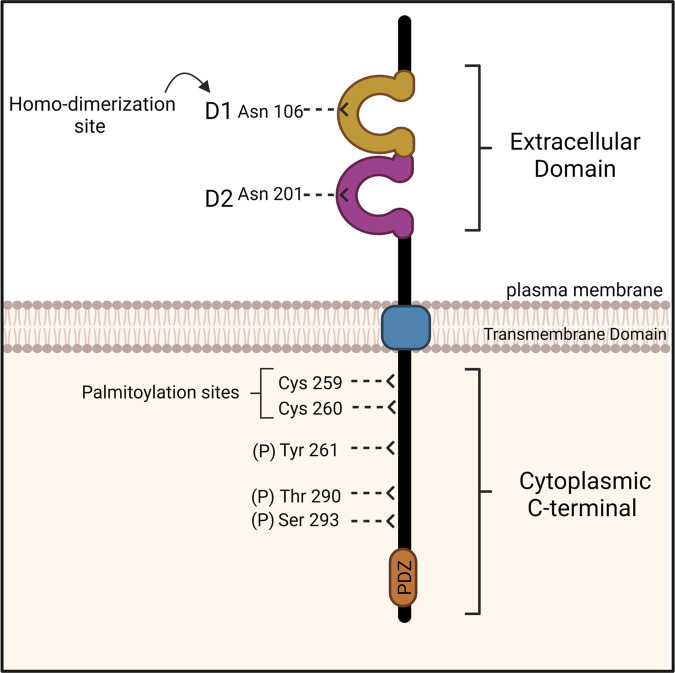
Structure of coxsackie and adenovirus receptor (CAR). CAR structure consists of two Ig-like extracellular domains D1 and D2 each containing a glycosylation site (Asn106 and Asn201, respectively), a transmembrane domain (blue), and a cytoplasmic C-terminal. The cytoplasmic tail of CAR has three phosphorylation sites (Thr290, Ser293 and Tyr261) and two palmitoylation sites (Cys259 and Cys260). The C-terminus also contains PDZ binding domain mediating intercellular protein binding. Figure adapted from [3] and created with BioRender.com.

The *CXADR* gene is located on chromosome 21 (21q11.1) encoding for a 365 amino acid protein [8]. CAR is composed of two immunoglobulin (Ig) like extracellular domains, a transmembrane domain, and ∼120 amino acid unstructured cytoplasmic tail (Figure 1). The extracellular domain of CAR consists of N-terminal domain (D1) and is connected to a membrane proximal domain (D2). The cytoplasmic tail has two characterised phosphorylation sites, two palmitoylation sites, and a class-I PDZ binding motif at the C-terminus [3]. CAR is phosphorylated on at least two residues, Serine 290, and Threonine 293 by Protein Kinase C δ (PKCδ; Figure 1) and phosphorylation of these residues leads to destabilisation of CAR at the plasma membrane of epithelial cells [9–11].

In the epithelium, CAR is predominantly localised to tight and adherens junctions where it can homodimerise through interactions between the D1 and D2 domains. CAR forms a complex with intracellular molecules to enable functional effects at these sites (summarised in Table 1). The intracellular interactions are in part enabled by the PDZ motif, forming complexes with PDZ-domain containing adaptor proteins, including ZO-1, MUPP-1, MAGI-1, PIST and LNX [12–16]. CAR can associate with F-actin via complexes formed with ZO-1 and MUPP-1, and this contributes to co-ordination of cytoskeletal dynamics [3,13]. CAR was also recently shown to form a complex with the focal adhesion kinase (FAK), the non-receptor tyrosine kinase Src and adhesion scaffold protein paxillin that may contribute to CAR-dependent control of the GTPase Rap1 and β1 integrin activation, resulting in enhanced cell–matrix adhesion [17]. CAR is further proposed to bind to tubulin in human glioma cells which results in reduced migration through microtubule stabilisation [18]. CAR also plays a role in adherens junctions (AJ) stability by promoting endocytosis of E-cadherin in bronchial epithelial cells [10,11]. Upon adenoviral infection, CAR also associates with β-catenin which further disrupts E-cadherin localisation and clustering [10]. LNX-2 was also identified to form a complex with CAR, which was recently shown to contribute to its function in response to stress by activating the Notch signalling pathway and potentially regulating cell fate [19,20]. CAR also forms complexes with additional proteins such as YAP-1 and CAV1, with yet unknown functional relevance [9], but as these are both mechano-regulated proteins, this raises an interesting possibility that CAR can also act as a sensor of external mechanical forces.

**Table 1:** Epithelial CAR interactions and binding partners

Binding partner	Context	Function of CAR complex	Refs
**Extracellular**			
Fibronectin	§ *in vitro*	Unknown	[23]
Agrin	§ *in vitro*	Unknown	[23]
Tenascin-R	§ *in vitro*	Unknown	[23]
Coxsackie and adenovirus receptor	§ NIH3T3 § Chicken embryonic derived neural cells	Homodimer; cell–cell adhesion	[23]
Junction adhesion molecule C	§ Mouse testis	Unknown	[60]
Junction adhesion molecule L	§ CAR expressed in epithelial (T84) and endothelial (HMEC-1) cells	Leukocyte transmigration	[23,28]
**Intracellular**			
Yes-associated protein -1	§ Human bronchial epithelial cells (16HBE)	Unknown	[12]
Caveolin-1	§ Human bronchial epithelial cells (16HBE)	Unknown	[12]
Tubulin	§ Human glioblastoma cells (U87 MG)	Reduces cell migration	[20]
Zonula Occludens - 1	§ T84, CALU-3, 16HBE and MDCK cells	Tight junction stability	[3]
Membrane-associated guanylate kinase	§ COS-7 cells	Tight junction stability	[15]
Ligand of numb protein	§ Human embryonic kidney 293 cells (HEK-293)	Tight junction stability	[17]
Multi-PDZ protein-1	§ Human epithelial cells (Caco-2)	Tight junction stability	[16]
Ligand of numb protein X-2	§ Embryonic mouse tissue	Stimulates Notch signalling	[21]
E-cadherin	§ Human bronchial cells (HBEC)	Cadherin endocytosis; adherens junction stability	[13]
β-catenin	§ Human bronchial cells (HBEC)	Cadherin endocytosis; adherens junction stability	[13]
Src proto-oncogene tyrosine protein kinase	§ *in vitro*	E-cadherin endocytosis; focal adhesion assembly	[13,20]
Focal adhesion kinase	§ *in vitro*	Unknown	[20]
Paxillin	§ *in vitro*	Unknown	[20]
β1 integrin	§ *in vitro*	Focal adhesion assembly	[12,20]
Protein interacting with protein C kinase	§ COS-7 cells	Tight junction stability	[15]

Table outlining reported extracellular and intracellular CAR-associated proteins and functional roles.

In addition to binding to viruses and forming homodimers *in trans*, epithelial CAR can also heterodimerise with members of the IgSF family including JAM-L and -C that are expressed on the surface of leukocytes including neutrophils and γδT cells (Table 1) [21–23]. These heterodimeric interactions facilitate leukocyte trans-epithelial migration and T cell activation, respectively [9,24–26]. The D2 domain of CAR was also observed to facilitate binding with different extracellular matrix proteins including fibronectin, agrin or tenascin-R in biochemical assays [21], but the nature of this binding in cells and contributions to CAR-dependent functions remain unclear.

## CAR contributions to tissue homeostasis

Deletion of CAR in the germline in mice causes early embryonic lethality, primarily due to loss of CAR from intercalated disks, and loss of electrical conductance between cardiomyocytes resulting in heart failure [27–29]. CAR is also highly expressed in the brain during development and neuronal-specific deletion of *CXADR* leads to defects in adult neurogenesis and synaptic function [30]. CAR additionally plays an important role in liver, skeletal muscle and the lymphatic system development, and targeted deletion in adult mice leads to dilated intestinal tract, atrophy of the exocrine pancreas, and abnormal thymopoiesis [30,31]. CAR is expressed at very low levels in the endothelium of vessels in intact heart tissue but is locally up-regulated following myocardial infarction in subsets of cardiac CD31+ cells, suggesting pathological settings contribute to fine-tuning CAR levels to elicit downstream functional responses [24]. CAR overexpression in cardiac myocytes also leads to disrupted adherens junctions *in vivo* inducing cardiomyopathy, further demonstrating that the correct balance of CAR expression is important in tissue homeostasis [25].

CAR in epithelial cells can control junctional protein dynamics and this is regulated through phosphorylation of the CAR C-terminus at S290/T293 by PKCδ [3,17]. Pro-inflammatory cytokines contribute to trans-epithelial migration of leukocytes and have also been shown to mediate JAM family protein localisation and function [10]. Treatment of cells with the pro-inflammatory cytokine TNFα results in an increase in phospho-CAR levels in lung epithelial cells *in vitro* and *in vivo* and this requires CAR homo-dimerisation *in trans* [23]. In both human and mouse lung epithelium, CAR promotes immune cell recruitment in response to house dust mite allergen in part due to release of pro-inflammatory cytokines from epithelial cells [9]. Interestingly, deletion of CAR from the lung epithelium also leads to increased matrix remodelling under basal conditions, in part due to loss of barrier function that drives enhanced release of epithelial TGFβ. This in turn leads to increased contractility and extracellular matrix (ECM) production by fibroblasts and smooth muscle cells [9]. These findings collectively highlight epithelial CAR as an emerging important regulator of the interplay between epithelial tissues and stromal/immune compartments.

## CAR contributions to tumour growth

**Figure 2.**
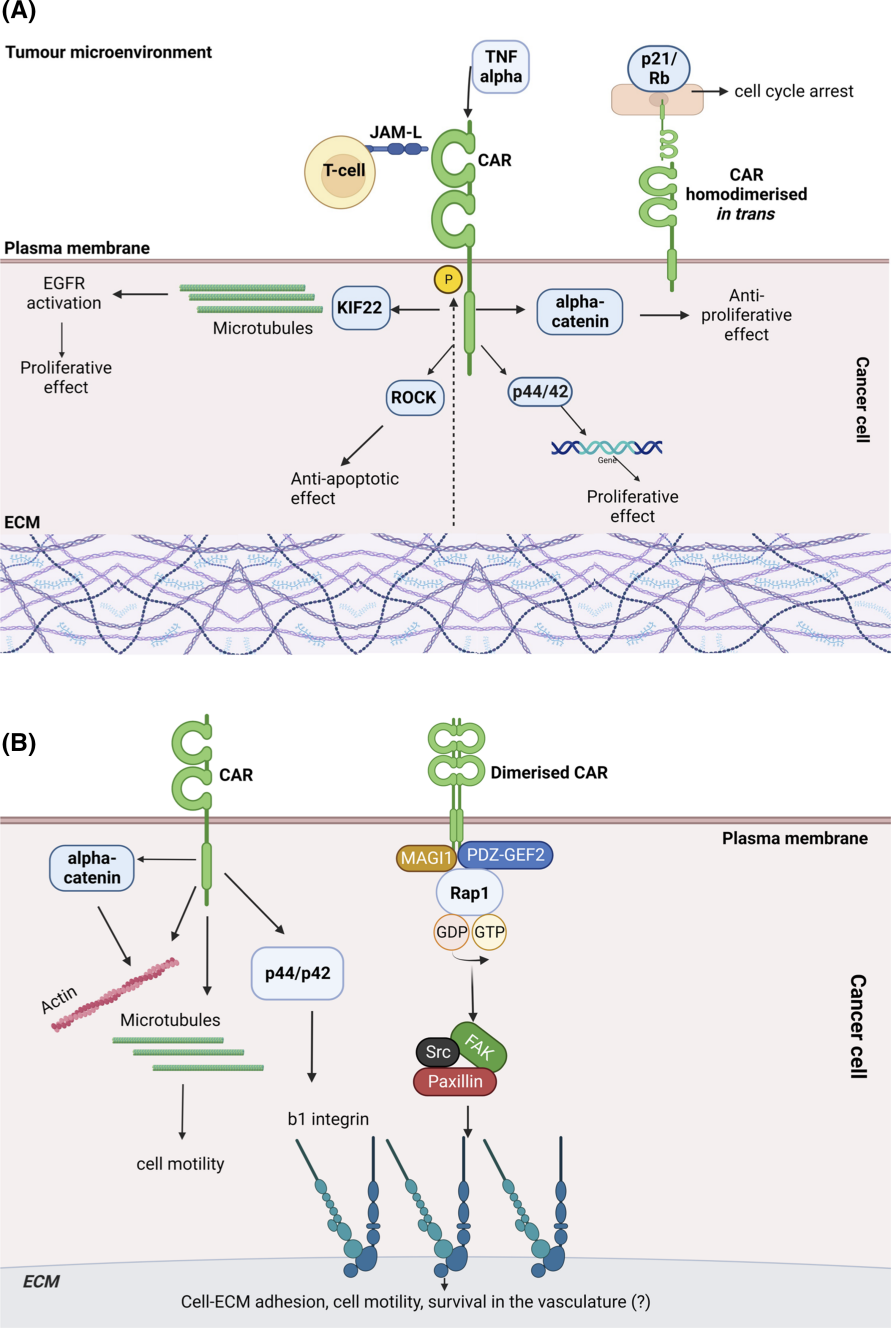
Proposed roles for CAR in cancer. (**a**) Roles for CAR in cancer cell proliferation: CAR has been shown to play a controversial role in cancer cell proliferation. CAR promotes cancer cell proliferation via interaction with microtubule-associated protein KIF22 and ROCK. Conversely, CAR inhibits cancer cell proliferation via suppression of alpha-catenin expression and accumulation of p21 and hypo-phosphorylated retinoblastoma (Rb) protein when it undergoes homodimerization in trans. CAR phosphorylation influences its role in cancer cell proliferation. CAR can be phosphorylated in response to TNF produced in the tumour microenvironment or in response to changes in ECM stiffness. Phospho-CAR can increase p44/42 activation resulting in changes in gene expression and cell proliferation. Interactions of CAR with JAM-L expressed on immune cells may influence the role of CAR in cancer cell proliferation. (**b**) Roles for CAR in cancer metastasis: CAR has a dual role in cancer metastasis. CAR contributes to cancer metastasis via regulation of cell–ECM adhesion mediated by beta integrins. CAR may promote survival of cancer cells in the vasculature via beta integrins. CAR influences cell motility via interactions with α-catenin, actin and microtubules.

Given the importance of CAR in mediating cell adhesion and stromal cell behaviour, several studies have examined potential roles for CAR in tumourigenesis (summarised in Figure 2). These studies have revealed that CAR expression levels vary in different types of cancer and its role in tumour progression remains controversial [32]. For example, CAR promotes cell proliferation in lung cancer cells and oral squamous carcinoma cells [33–35]. shRNA depletion of CAR leads to a significant reduction in EGF-dependent proliferation of human lung cancer A549 cells *in vitro* and siRNA CAR-depleted H1975 cells injected subcutaneously in immunocompromised mice form smaller tumours [33]. The same study demonstrates that CAR enables A549 lung cancer cell proliferation through binding to the microtubule associated protein KIF22 that promotes activation and delayed internalisation of Epidermal Growth Factor Receptor (EGFR; [33]). Silencing CAR expression in non-small-cell lung cancer cells NCI-H1703 via transfection with CAR antisense oligonucleotides leads to reduced xenograft formation in *scid/scid* mice subcutaneously injected with these cells [34]. Similarly, CAR knockdown by siRNA in HSC-2 oral squamous carcinoma cells results in reduced proliferation due to cell–cell dissociation, accompanied by cytoplasmic translocation of junctional E-cadherin *in vitro,* and a decrease in tumour growth *in vivo* in ectopic-xenograft mice models upon intraperitoneal injection of siCAR HSC-2 cells [35]. CAR has also been suggested to promote breast cancer survival in a murine mammary cancer model as adenocarcinomas developed upon syngeneic implantation of preneoplastic mammary tissue show increased CAR and bcl-2 expression compared with non-invasive precursor lesions. CAR-overexpressing Hela, CaSki and A2780 cancer cell lines also display enhanced cell survival upon application of tumour necrosis factor-related apoptosis-inducing ligand and this is accompanied by reduced activation of caspase 3 and higher expression of bcl-2 or bcl-XL, depending on the cell line [36]. Immunoprecipitation assays of CAR-transfected HCS-2 oral squamous cell carcinoma cells showed that CAR associates with Rho kinase ROCKI and ROCKII resulting in inhibition of ROCK activity. The same study suggested that CAR promotes growth through suppression of apoptosis downstream of ROCK [35]. CAR siRNA knockdown also results in reduced cell proliferation in colon cancer DLD1 cells. This may be associated with reduced α-catenin expression observed after CAR down-regulation, as enhanced growth of CAR-depleted cells can be restored through ectopic expression of α-catenin [37].

Conversely, other reports have shown that CAR inhibits cell proliferation in glioma cells, gastric, bladder and prostate cancers *in vitro* [38–41]. The reported mechanisms appear to differ depending on the tumour type and experimental model. One study suggests that CAR acts as a tumour suppressor in glioma as U-118 MG glioma cells transfected with hCAR vector and subcutaneously injected in nude mice form smaller tumours. In this context, the extracellular D2 domain of CAR is not required for CAR to inhibit tumour growth or formation of colonies by U-118 MG cells, however the mechanisms by which CAR acts as a tumour suppressor were not investigated [39]. Higher CAR expression in PC3 prostate cancer cell sublines correlates with reduced cell proliferation *in vitro* and formation of smaller tumours upon subcutaneous injection into nude mice [40]. Similarly, reduced CAR mRNA in human bladder cancer specimens is observed suggesting that higher CAR expression in CAR-transfected bladder cancer T24 cells correlates with reduced cell proliferation *in vitro* [41]. Loss of CAR is also correlated with more severe disease states in gastric cancer. *In vitro* experiments assessed the role of CAR in proliferation, migration and invasion of AGS, MKN45 and MKN28 gastric carcinoma cells. Down-regulation of CAR by siRNA in AGS cells enhanced cell proliferation, migration in a chemotaxis chamber and invasion into Matrigel. In contrast, CAR overexpression in MKN45 and MKN28 cells resulted in reduced cell proliferation, migration and invasion, although the mechanisms underpinning this are yet to be explored [38].

The effect of CAR on tumour cell growth also appears to vary depending on CAR expression levels. CAR overexpression moderately reduces cell proliferation in CMT167 mouse lung cancer cells with high endogenous CAR expression but does not affect growth in those with low endogenous CAR (LLC1 cells) [17,42], indicating that other factors are required to synergise with CAR to promote proliferation. Blocking formation of CAR-mediated cell–cell contacts using the RmcB monoclonal antibody reduces the CAR-growth inhibitory effect and it elicits lower p21 levels and hyper-phosphorylated retinoblastoma accumulation in bladder cancer T24 cells, [41]. This suggests that CAR-dependent effects on cell proliferation may require initial CAR homodimerisation *in trans* across cell–cell junctions in solid tumours, but that a cycle of phosphorylation of CAR is required to enable cell adhesion dynamics to facilitate growth.

Differences in the *in vitro* and *in vivo* model systems used in previous studies may provide some explanation for the apparent discrepancies in findings. Cells *in vivo* are more likely to form cell–cell contacts within solid tumours, which could favour CAR-dependent homodimerization and subsequent signalling compared with 2D cultures. Indeed, although CAR can suppress lung cancer cell proliferation *in vitro*, it promotes tumour growth in the same cells (CMT167 cells) *in vivo* upon subcutaneous injection in immunocompetent mice [17]. CAR expression levels and localisation have been shown to differ between 2D and 3D environments, suggesting that changes in the tumour microenvironment could affect CAR-dependent signalling. Anders *et al.* compared CAR expression and localisation in normal breast epithelial cells (S1) and their malignant derivative (T4-2) breast cancer cells grown on either tissue culture plastic (2D) or in 3D in Matrigel. Although CAR expression level does not differ between S1 and T4-2 cells when cultured in 2D, CAR levels are strikingly reduced in S1 cells compared with T4-2 cells in 3D cultures. Furthermore, CAR localisation changes from cell–cell junctions to the cytoplasm in T4-2 cells cultured in 2D or 3D, respectively [43].

Changes in matrix stiffness are commonly observed in tumours and can lead to changes in cancer cell behaviour. We recently demonstrated that CAR phosphorylation increases with substrate stiffness in LLC and CMT lung cancer cells grown on 1.5 kPa, 28 kPa plates and glass coverslips (>1 GPa) and this could contribute to CAR-dependent effects on tumour growth *in vivo* [17]. Similarly, the CAR-related family member JAM-A has recently been reported to enhance tight junction stability in MDCK cells on higher stiffness surfaces as its junctional recruitment is regulated by ECM stiffness [44]. This implies that mechanical forces exerted through integrin-based adhesions act in synergy with cell–cell contact receptors to drive differential signalling. Moreover, as noted previously, CAR has recently been shown to form complexes with mechano-sensitive proteins YAP and caveolin in human bronchial epithelial cells (16HBE) [9]. Exploring potential roles of the mechanical environment within tumours in controlling CAR expression, localisation and post-translational modification represents an important future research avenue to fully understand how environmental cues, both mechanical and chemical, contribute to CAR function.

Other signals from the tumour microenvironment could also explain the differences between *in vitro* and *in vivo* studies, and data in other tissue or pathological settings may provide insight into this. CAR has been shown to interact with the Junctional Adhesion Molecule-Like protein, JAM-L, expressed on neutrophils and γδ T cells [26,45–47]. CAR expressed on keratinocytes binds to JAM-L protein expressed on epithelial γδ T cells (DETC). This binding induces cytokine and growth factor production, MAP kinase pathway activation and DETC cell proliferation during tissue repair [26,46]. CAR expression is up-regulated in keratinocytes adjacent to the wound and blockade of JAM-L-CAR interaction upon epidermal wounding impairs activation of DETC at the wound edge and causes a reduced healing response [46]. CAR expressed on gut epithelial cells (T84 cells) interacts with JAM-L on neutrophils to facilitate neutrophil trans-epithelial migration commonly observed in inflammation [45]. Similarly, CAR phosphorylated by TNFα promotes efficient trans-epithelial migration of monocyte-derived THP-1 cells and neutrophils across human bronchial epithelial cell (HBEC cells) monolayers *in vitro* and into the bronchial lumen in mouse models *in vivo* [11].

Allergen-induced CAR phosphorylation leads to transient destabilisation of CAR cell–cell adhesions, release of pro-inflammatory cytokines from the epithelium and enhanced neutrophil and γδ T cell recruitment to the lung. Upon exposure to the pro-inflammatory allergen house dust mite (HDM), immune profiling of cells within the lungs of a mouse model lacking CAR in the respiratory epithelium, showed a reduction in neutrophil and γδ T cell infiltration [9]. Supernatant from HDM-challenged CAR-depleted 16HBE human bronchial epithelial cells shows a reduced ability to drive chemotaxis of HL60 neutrophils. Furthermore, cytokine array analysis of supernatants from CAR-depleted HDM-challenged lung epithelial cells show a significant reduction in secretion of pro-inflammatory cytokines. Interestingly, supernatant from HDM-treated CAR-depleted cells leads to up-regulation of the immunosuppressive cytokine IL-10, suggesting CAR may regulate cytokine production to affect immune infiltration. CAR depletion leads to activation of the GSK3-β:SMAD2/3 signalling axis and TGFβ signalling, which in turn increases ECM deposition and this could represent an additional way for CAR to regulate immune cell recruitment [9].

CAR has also been shown to contribute to tumour progression via regulation of anti-tumour immunity. JAM-L expressed in K562 myeloid leukaemia cells, transfected with a vector encoding JAM-L, promotes leukocyte adhesion to endothelial cells by interacting with CAR, as the leukocyte-endothelial cell interaction is abrogated by addition of CAR-Fc chimeric molecules. This study also shows that JAM-L-CAR interactions may have different relevance in different types of leukocytes. For example, human neutrophils and monocytes adhere to CAR Fc protein *in vitro*, but addition of JAM-L Fc only abolishes adhesion of neutrophils, and not monocytes [47]. Heterodimeric interactions between CAR expressed on melanoma cells, with JAM-L on T cells, promotes CD8 and γδT cell infiltration. JAM-L knockout in a mouse B16F10 melanoma model leads to accelerated tumour growth caused by decreased CD8 and γδ T cell activation and tumour infiltration. This suggests that CAR loss, observed during advanced stages of melanoma, may cause a reduction in JAML-CAR interaction-induced anti-tumour immunity mediated by CD8 and tumour-associated γδ [48]. Thus, CAR may promote tumour growth *in vivo* through interactions with specific subtypes of immune cells within the tumour microenvironment. However, additional studies are required to fully elucidate mechanisms underlying CAR-induced tumour immune infiltration.

Phosphorylation of CAR cytoplasmic tail in MCF7 human breast cancer cells was shown to be associated with increased activity of p44–p42 MAPK, reported to regulate cell proliferation [49,50]. Therefore, TNFα-induced CAR phosphorylation, observed in bronchial epithelial cells [11] could also promote CAR-induced p44/p42 MAPK activation and possibly alter cell proliferation . CAR expression in airway epithelial cells can also be enhanced by interleukin-8 (IL-8), a pro-inflammatory cytokine released by macrophages and tumour cells [51]; this potentially important modulator of CAR function in cancer would be interesting to explore in future studies. Indeed, the temporal and spatial control of CAR transcription in tumours remains poorly understood, but reports highlight transforming growth factor-β (TGFβ), ZEB1 and the transcription factor Sp-1 as potential regulators of expression levels in cancer [52–54]. CAR mRNA and protein expression in HUVEC endothelial cells is reduced upon exposure of the cells to pro-inflammatory cytokines such as TNFα and IFNγ [55]. Thus, CAR has been shown to play a dual role in cancer cell proliferation and this function varies according to tumour type, microenvironment, CAR expression level and experiment setting. However, key knowledge gaps exist in understanding how the tumour microenvironment influences CAR expression, post-translational modification and function, and these represent important future goals to understand these discrepancies in findings.

## CAR-dependent control of cancer cell metastasis

CAR has been implicated in several steps of the metastatic cascade. The latter is a multi-step process which includes detachment from primary tumour, local invasion, intravasation and survival in the vasculature, extravasation and finally colonisation at a distant secondary site [56]. Regulation of cell–ECM adhesion is required to enable cell invasion. CAR and other JAM family proteins have been shown to regulate cell motility via co-ordination of integrin activity [57]. Integrins integrate extracellular and intracellular events via bi-directional signalling between the ECM and the actin cytoskeleton [58]. JAM-A dimerization in the epithelium enhances migration by binding to Afadin and the guanine nucleotide exchange factor RAP GEF2 to activate Rap1A, thereby stabilising β1 integrin activity [59]. As has been shown for JAM-A, CAR overexpression results in increased activation of the GTPase Rap1 leading to enhanced β1 integrin activation in lung cancer cells [17]. CAR may regulate Rap1 activity by creating a complex with MAGI-1 and PDZ-GEF2, and like JAM-A, this complex could be formed through association of proteins with the CAR PDZ-binding domain. CAR has been shown to form a complex with focal adhesion proteins FAK, Src and paxillin in LLC and CMT mouse lung cancer cells and these molecules may be recruited by CAR to regulate cell–ECM adhesion [17]. As CAR does not translocate to focal adhesions, it may exert control over β1 integrin activity from cell–cell junctions or an intracellular compartment. CAR can also regulate focal adhesions through p44/p42 MAPK activation resulting in increased β1 and β3 integrin activity in MCF7 human breast cancer cells [50].

Adhesion molecules are known to be critical regulators of cell migration and invasion [59–61]. However, conflicting roles for CAR in these processes have also been reported. CAR suppresses migration and invasion in gastric and lung cancer cells [17,38] and overexpressing CAR reduces migration and invasion in ovarian and cervical cancer cells [36] and glioma cells cultured as spheroids [62]. Conversely, CAR may enhance the invasive behaviour of lung cancer cells by promoting epithelial–mesenchymal transition (EMT). EMT is a phenotypic shift that occurs in progression of some tumours whereby cells adopt reduced epithelial characteristics (such as E-cadherin expression) and increase mesenchymal markers (such as vimentin). Cells expressing high CAR display spindle-shaped morphology, whereas low CAR expression is associated with lung cancer cells presenting an epithelial phenotype [42]. Conversely loss of CAR can lead to increased breast cancer cell sensitivity to TGFβ1-induced EMT via hyperactivation of AKT [63]. Moreover, manipulating CAR expression in two different mouse lung cancer cell lines (CMT and LLC cells) led to altered invasion but did not change expression levels of typical EMT markers [17]. Interestingly, CAR overexpression and CRISPR-mediated depletion both promote cell motility in these mouse lung cancer cells [17]. This suggests that other mechanisms might be activated upon CAR depletion. Indeed, CAR depletion in DLD1 colon cancer cells enhances cell migration and invasion into Matrigel and leads to reduced α-catenin expression. However, ectopic re-expression of α-catenin in CAR-depleted cells only partially restores basal levels of cell invasion, therefore this pathway requires further investigation [37]. α-catenin regulates actin assembly [64] and CAR forms a complex with actin in pull down assays from mouse brain lysates, [65] providing potential additional mechanisms through which CAR can directly influence cytoskeletal dynamics in cell migration and invasion. CAR has also been shown to inhibit U87 glioma cell migration by reducing microtubule dynamics [18] which may additionally contribute to sensitivity to the chemotherapy agent Taxol, derivatives of which are in common clinical use for cancer. In this study, CAR was shown to interact with tubulin and microtubules using immunoprecipitation assays. Furthermore, CAR increases U87 cell sensitivity to the microtubule stabilising agent paclitaxel, possibly by enhancing microtubule bundling as observed in microtubule binding affinity assays [18].CAR overexpression in lung cancer cells promotes adhesion to the lung upon intravenous injection in immunocompetent mice. Moreover, CAR overexpressing lung tumour tissue sections, obtained upon intravenous injection of lung cancer CMT cells in immunocompetent mice, show higher β1 integrin activation suggesting that CAR might promote adhesion to the lung via regulation of integrins [17]. β1 integrins promote tumour cell arrest in the pulmonary vasculature via interactions with laminin present in the exposed basement membrane [66]. Conversely, CAR acts as a metastatic suppressor as CAR expression reduces accumulation of melanoma cells in the lung upon intravenous injection potentially due to CAR-induced reduction in αv, α4, β3 and β1 integrin expression [67]. In agreement with this, CAR expression decreases in bladder tumours at stages 3/4 compared with stage 1, suggesting that loss of CAR is required for haematogenous spread of this type of cancer [41]. Metastatic cells rely on integrins to survive in the vasculature and colonise a secondary site [68]. We speculate that CAR-dependent β1 integrin activity may also promote cell survival in the vasculature and upon seeding of metastatic cells within distant organs. In this respect, CAR may also behave in a similar manner to E-cadherin, which has recently been shown to act as a survival factor in systemic dissemination and colonisation phases of metastasis [67].

## CAR as a therapeutic target for cancer

CAR represents a potential therapeutic target given its pro-tumorigenic roles in tumour growth, adhesion, and metastasis. However, heterogenous CAR expression in different types of cancer, stages of tumour progression and normal tissues should be considered when developing a CAR-targeting anti-cancer treatment. CAR expression levels have been shown to differ depending on the stage of cancer progression. For example, up-regulation of CAR has been implied to promote carcinogenesis in early-stage breast cancer and precursor cells [36]. In this study, a syngeneic preneoplastic mammary tissue implant mouse model was used to show that CAR expression is up-regulated in invasive adenocarcinomas compared with precursor non-invasive lesions [36]. However, CAR is down-regulated in advanced disease stages in several tumour types displaying loss of differentiation [38,69–72]. Compared with healthy mucosa, CAR mRNA expression is increased during early carcinogenesis in colon adenomas and decreased during cancer dissemination in colon cancer metastases based on RT-PCR analysis of human tissue samples [73]. CAR expression can be up- or down-regulated during tumour progression to promote different aspects of the metastatic cascade. For example, CAR mRNA expression is higher during early stages of mouse and human melanoma, but this significantly decreases as the tumour progresses when CAR-promoted tumour immunity becomes disadvantageous for cancer progression [48]. Therefore, it would be of crucial importance to determine the right tumour stage for any potential anti-CAR therapy administration through careful histological and mRNA analysis of the cancer tissue.

Targeting CAR also presents a challenge due to potential toxicity from targeting normal tissues. For example, mouse-human chimeric ch6G10A antibodies against CAR (targeting the D2 membrane proximal extracellular domain) inhibited tumour growth and metastatic formation of prostate cancer and small cell lung cancer upon subcutaneous injection of DU-145 or NCI-H69 cells or orthotopic implant of highly metastatic DMS273 cells into nude mice[74]. However, the same study showed that biotinylated anti-CAR 6G10A antibody reacts with CAR present on normal skin, prostate, and kidney tissues in tissue immunostaining experiments [74]. This raises some concerns about off-target effects, which are currently unknown for anti-CAR therapies. Given the frequent overexpression of CAR seen in different cancer subtypes, the potential of CAR as an oncolytic adenoviral therapy target has also been considered [72,75]. In this context, CAR that is highly expressed in tumour cells is used as a receptor for entry of viruses to drive tumour cell death with a high degree of specificity. For example, CAR has been successfully used as a receptor for delivery of a telomerase-specific oncolytic virus (OBP-301) in treatment of radioresistant oral squamous cell carcinoma [76]. Interestingly, chemotherapy has been shown to enhance CAR expression in one study whereby subsequent use of oncolytic therapy increased death of chemotherapy-resistant breast cancer cells [77]. However, it is notable that human erythrocytes express high levels of CAR leading to issues with systemic delivery and reduced virus reaching the tumour [78]. Furthermore, oncolytic therapy would not be useful in the case of low CAR-expressing tumours which could possibly be those that have undergone EMT in more progressive tumour stages [52], highlighting the importance of careful profile of CAR expression prior to oncolytic therapy. Additional analyses are also needed to determine potential side effects, dosing and toxicity of anti-CAR treatments or oncolytic viruses for cancer therapy in future.

CAR has been shown to regulate immune responses in epithelial cells through direct interactions with leukocyte surface receptors leading to enhanced immune cell adhesion and trans-epithelial migration [79]. These findings raise the possibility that CAR might play a role in tumour-immune cell infiltration. However, the role of CAR in immune cell recruitment in cancer has not been studied to date. Epithelial CAR interacts with JAM-L on neutrophils and γδT cells [11,22,55] . Tumour infiltration by CAR-interacting neutrophils and γδT cells can be associated with pro-tumorigenic effects, therefore blocking CAR or the phosphorylation of CAR may be beneficial in blocking immune cell-promoted tumour progression [80]. For example, neutrophils produce a cytokines and matrix-remodelling proteinases that promote tumour cell growth and invasiveness [80]. γδT cells have also been shown to promote tumour growth via secretion of IL-17 and can also inhibit anti-tumour function of other immune cells such as CD8+ T cells [81]. However, tumour-infiltrating neutrophils and γδ T cells can also have anti-tumorigenic effects, and the ability of CAR to recruit these cells could be exploited to reduce tumour growth [82]. Despite being accepted as pro-tumorigenic, tumour-infiltrating neutrophils can have a dual nature, and anti-tumour N1 and pro-tumour N2 subsets have been defined and are modulated by TGFβ [83]. Similarly, γδ T cells can also promote anti-tumour response of adaptive immune cells or kill tumour cells directly [81]. Thus it would be important to focus future studies on characterisation of immune cell subtypes that correlate with high CAR expression in disease and further determine potential contributions from tumour-expressed CAR to controlling these subtypes.

Targeting CAR with anti-CAR monoclonal (anti-D1) and polyclonal (anti-D1 and anti-D2) antibodies blocks neurite attachment and growth, whereas Ad5FK promotes these activities [21]. Additionally, the D2 domain is required for CAR heterophilic interactions with ECM glycoproteins, whereas both D1 and D2 domains are needed for homophilic interactions in neural cells. Therefore, targeting different parts of CAR ectodomains may lead to different biological outcomes. Future experiments using CAR blocking antibodies targeting D1 and/or D2 domains could be employed to investigate this further [21]. Moreover, as stabilisation of CAR at the plasma membrane of normal epithelial cells reduces migration and increases barrier integrity [9–11], therapeutic antibodies directed to stabilise CAR homodimers may provide an interesting route for consideration in future.

CAR could also be used as a target for gene therapy to enable entry of replication-defective adenoviruses that deliver therapeutic DNA into target tumour cells, such as p53 in non-small lung cell cancer [84]. However, given that depletion of CAR can lead to pro-tumorigenic effects, delivering oligonucleotides to remove CAR itself may result in deleterious effects, depending on the stage of the tumour. Highly expressed CAR present on the surface of tumour cells could also be used as a target antigen for chimeric antigen receptor T-cell therapy (CAR-T) to elicit an anti-tumour response. As for oncolytic therapy, this therapeutic approach could also lead to off-target toxicity in CAR-expressing non-tumoral cells. Therefore, it would be interesting to determine whether CAR expressed on tumour cells presents specific post-translational modifications that could be used to reduce off-target effects. On-target specificity could also be enhanced using tandem CAR-T cells for multiple target antigens. CAR could be used as a target antigen alongside a tumour-specific biomarker to enhance CAR-T cell specificity for tumour cells.

In summary, whilst CAR represents an interesting target for cancer therapy, more work is required to understand the context-specific roles for this receptor during tumour progression to determine feasibility of this approach.

## Perspectives

- Maintenance of cell–cell adhesion is critical for tissue homeostasis and CAR plays a key role in this process.
- CAR is a cell–cell adhesion protein that can also bind to surface receptors on leukocytes to promote inflammation. CAR is also dysregulated in cancer, although the functional relevance and potential therapeutic implications remain controversial.
- Understanding CAR function at different disease stages and contribution to the tumour immune microenvironment are important future avenues for further exploration.

## Abbreviations

CAR: coxsackievirus and adenovirus receptor
CAR-T: chimeric antigen receptor T-cell therapy
ECM: extracellular matrix
EMT: epithelial–mesenchymal transition
FAK: focal adhesion kinase
HBEC: human bronchial epithelial cell
IgSF: immunoglobulin superfamily
JAM: junction adhesion molecule
TGFβ: transforming growth factor-β
